# Supplementary material for: Zeolites as Adsorbents and Photocatalysts for Removal of Dyes from the Aqueous Environment
Source: Molecules. 2022 Oct 4;27(19):6582. doi: 10.3390/molecules27196582 (PMC9572986; doi:10.3390/molecules27196582)
Supplement: Supplementary file 1 [file molecules-27-06582-s001.zip › molecules-1897082-supplementary.pdf]

molecules

MDPI

SUPPLEMENTARY MATERIALS

Zeolites as adsorbents and photocatalysts for removal of dyes from the aqueous environment

Marina Rakanović [1], Andrijana Vukojević [2], Maria M. Savanović [2], Stevan Armaković [3], Svetlana Pelemiš [4], Fatima Živić [5,*], Slavica Sladojević [1], and Sanja J. Armaković [2,*]

[1] Faculty of Technology, University of Banja Luka, 78000 Banja Luka, Republic of Srpska, Bosnia and Herzegovina;
marina.rakanovic@tf.unibl.org (M.R.); slavica.sladojevic@tf.unibl.org (S.S.)
[2] University of Novi Sad, Faculty of Sciences, Department of Chemistry, Biochemistry and Environmental Protection, 21000 Novi Sad, Serbia; andrijana.vukojevic@dh.uns.ac.rs (A.V.); maria.savanovic@dh.uns.ac.rs (M.M.S.)
[3] University of Novi Sad, Faculty of Sciences, Department of Physics, 21000 Novi Sad, Serbia;
stevan.armakovic@df.uns.ac.rs
[4] Faculty of Technology Zvornik, University of East Sarajevo, 75400 Zvornik, Bosnia and Herzegovina;
svetlana.pelemis@tfzv.ues.rs.ba
[5] University of Kragujevac, Faculty of Engineering, 34000 Kragujevac, Serbia
* Correspondence: sanja.armakovic@dh.uns.ac.rs (S.J.A.); zivic@kg.ac.rs (F.Ž.); Tel.: +381-34-335-990 (F.Ž.); +381-21-485-2754 (S.J.A.)

Results and Discussion

Results of adsorption observation

Table S1. Summary overview of parameters for adsorption of RB from the aqueous environment on selected zeolites at two temperature

Zeolite	T [K]	$x / m \cdot 10^3$ [g/g]	$N \cdot 10^{-19}$	S (m^2)	Q (%) S / S_P	k	n	$\Delta_{ads}H_m$ [kJ/mol]
NH_4BETA	283	I=0.14<br>II=0.48<br>III=0.86	I=8.43<br>II=28.91<br>III=51.80	I=44.51<br>II=152.64<br>III=270.23	22.91	4.79	0.9740	-2.2917
	293	I=0.38<br>II=0.87	I=22.89<br>II=52.40	I=120.86<br>II=276.67	29.23	15.35	0.9090	-2.2143
NH_4ZSM-5	283	I=0.67	I=40.35	I=213.05	53.26	$7.91 \cdot 10^{-2}$	1.8769	-4.4161
	293	I=0.73	I=43.97	I=232.16	58.04	$4.99 \cdot 10^{-2}$	1.9048	-4.6401
NaY	283	I=0.30<br>II=0.49	I=18.07<br>II=29.51	I=95.41<br>II=155.81	13.96	$1.26 \cdot 10^{-2}$	1.8886	-4.4436
	293	I=0.25	I=15.06	I=79.52	8.84	$5.25 \cdot 10^{-2}$	1.2882	-3.1381

individual plateaus (N), total surface of adsorbed molecules on individual plateaus (S), the ratio of the surface of all adsorbed molecules and the specific surface of the zeolite (Q), Freundlich constant (k), dimensionless Freundlich intensity parameter (n), adsorption heat ($\Delta_{ads}H_m$)

Table S2. Summary overview of parameters for adsorption of MB from the aqueous environment on selected zeolites at two temperature

Zeolite	T [K]	$x / m \cdot 10^3$ [g/g]	$N \cdot 10^{-19}$	S (m^2)	Q (%) S / S_P	k	n	$\Delta_{ads}H_m$ [kJ/mol]
NH_4BETA	283	I=0.49<br>II=0.86	I=29.51<br>II=51.80	I=129.55<br>II=227.40	26.25	0.328	1.4516	-3.4154
	293	I=0.64	I=38.55	I=169.23	24.89	54.87	0.7321	-1.7834
NH_4ZSM-5	283	I=0.63	I=37.94	I=166.56	41.64	0.596	1.1519	-2.7103
	293	I=0.13<br>II=0.44	I=7.83<br>II=26.50	I=34.37<br>II=116.34	18.84	5.44	0.8675	-2.1132
NaY	283	I=0.29	I=17.47	I=76.69	8.52	0.109	1.2129	-2.8538
	293	I=0.097<br>II=0.44	I=5.84<br>II=26.50	I=25.64<br>II=116.34	7.89	0.190	1.0988	-2.6767

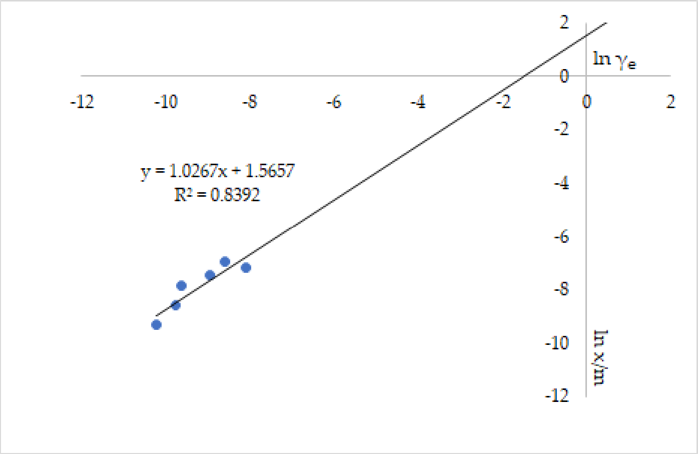

Figure S1. Functional dependence ln x/m of ln γ_e for the suspension RB-NH_4BETA at 283 K

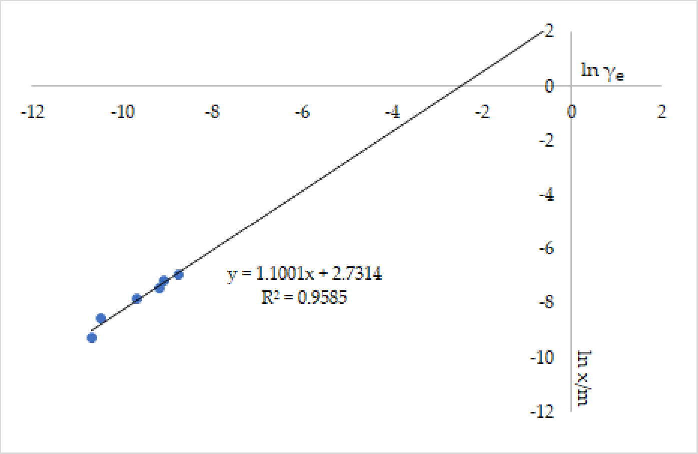

Figure S2. Functional dependence ln x/m of ln γ_e for the suspension RB-NH_4BETA at 293 K

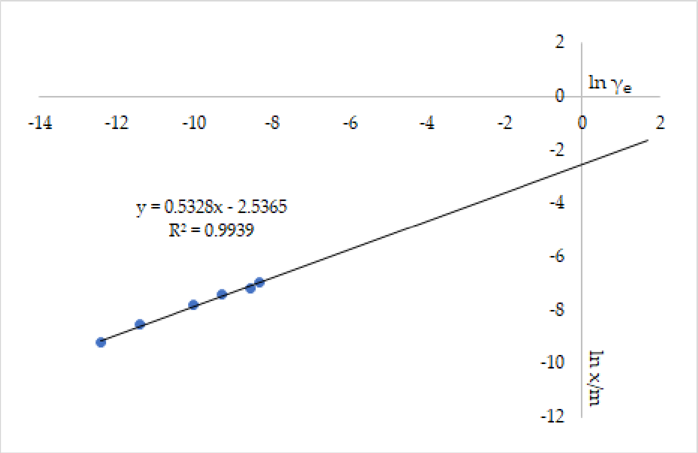

Figure S3. Functional dependence ln x/m of ln γ_e for the suspension RB-NH_4ZSM-5 at 283 K

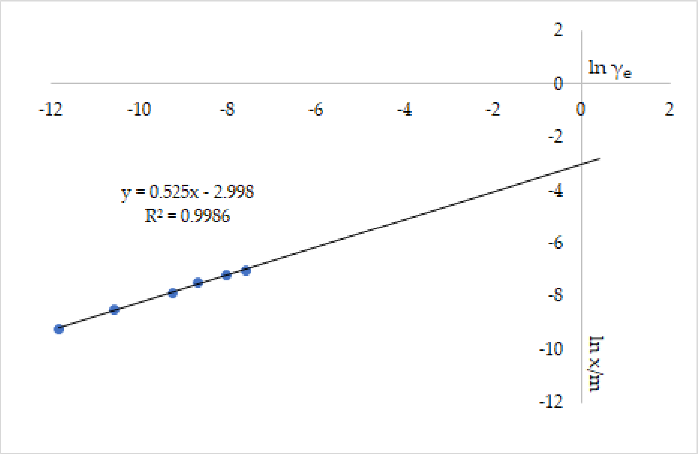

Figure S4. Functional dependence ln x/m of ln γ_e for the suspension RB-NH_4ZSM-5 at 293 K

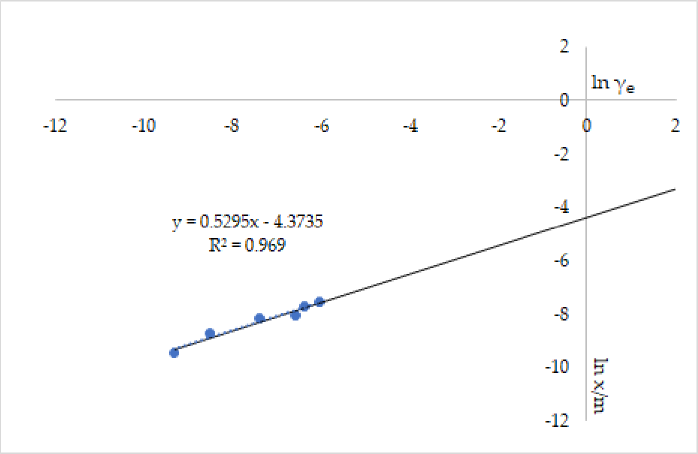

Figure S5. Functional dependence ln x/m of ln γ_e for the suspension RB-NaY at 283 K

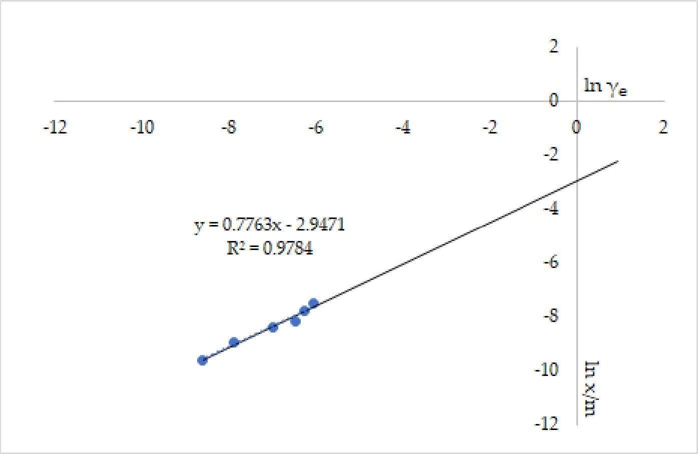

Figure S6. Functional dependence ln x/m of ln γ_e for the suspension RB-NaY at 293 K

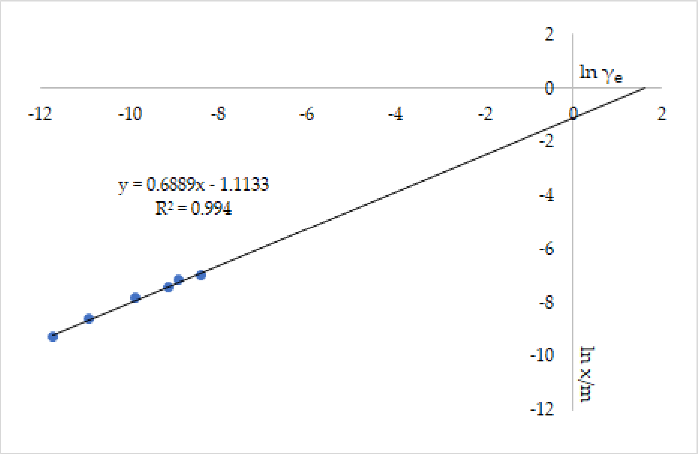

Figure S7. Functional dependence ln x/m of ln γ_e for the suspension MB-NH_4BETA at 283 K

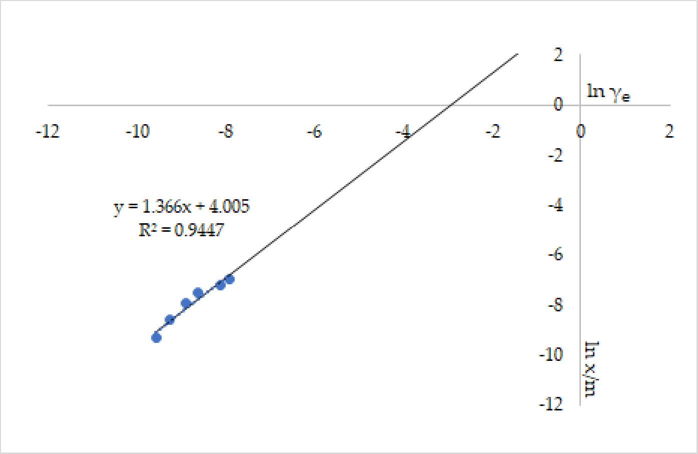

Figure S8. Functional dependence ln x/m of ln γ_e for the suspension MB-NH_4BETA at 293 K

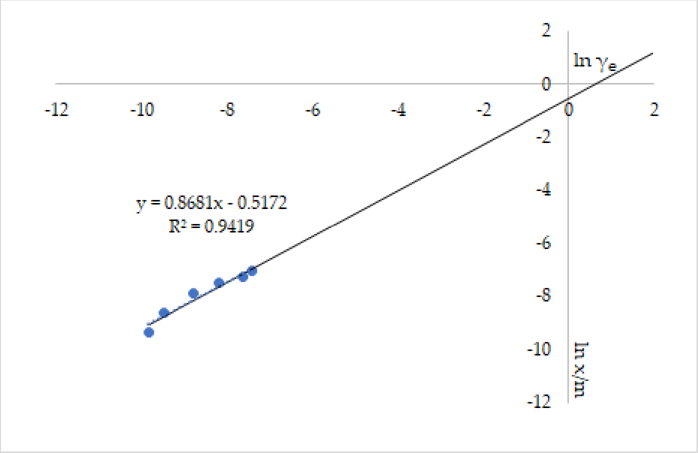

Figure S9. Functional dependence ln x/m of ln γ_e for the suspension MB-NH_4ZSM-5 at 283 K

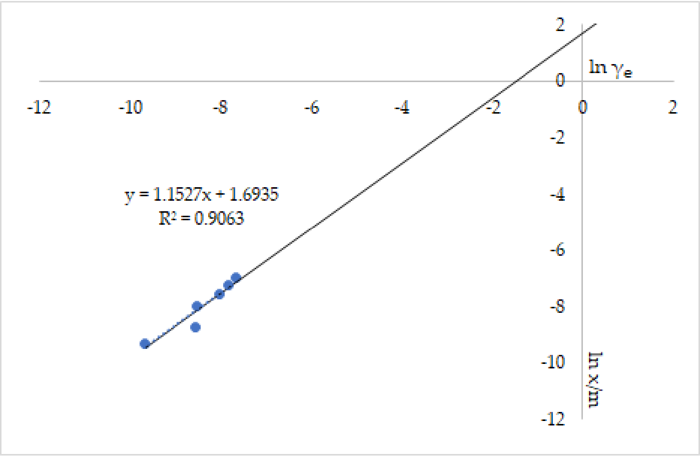

Figure S10. Functional dependence ln x/m of ln γ_e for the suspension MB-NH_4ZSM-5 at 293 K

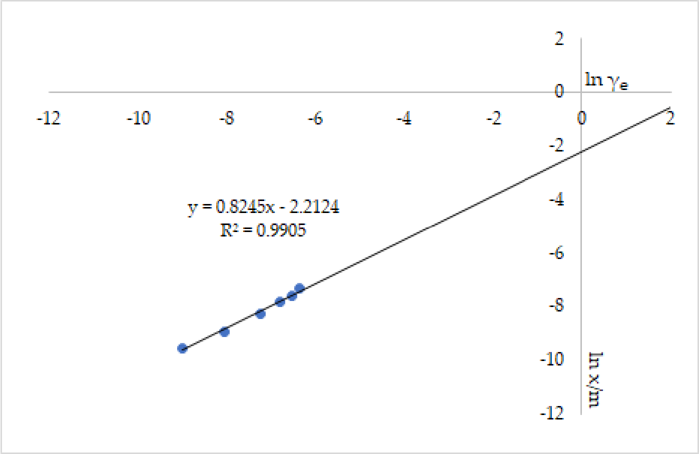

Figure S11. Functional dependence ln x/m of ln γ_e for the suspension MB-NaY at 283 K

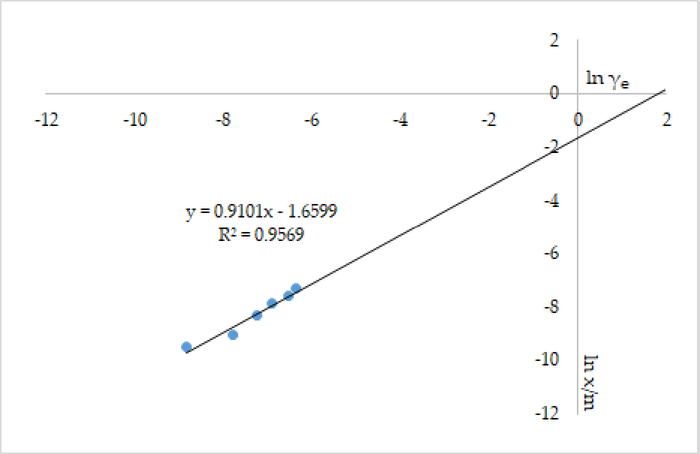

Figure S12. Functional dependence ln x/m of ln γ_e for the suspension MB-NaY at 293 K

Photodegradation

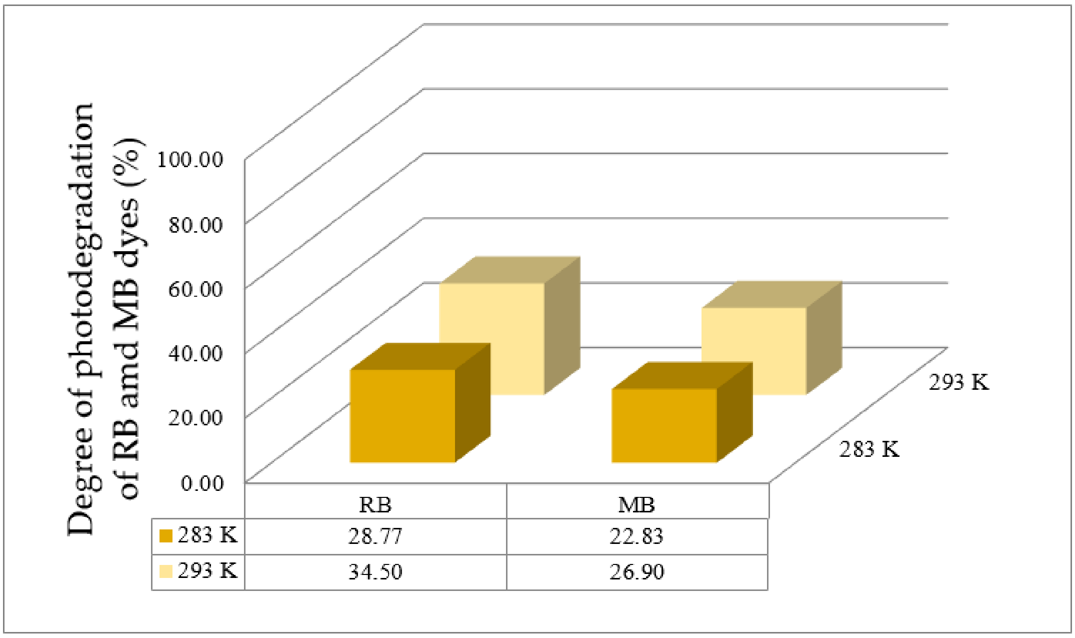

Figure S13. Degree of photolytic degradation of RB and MB dye under the UV radiation at two temperatures after 180 min radiation

Materials and Methods

Chemicals and solutions

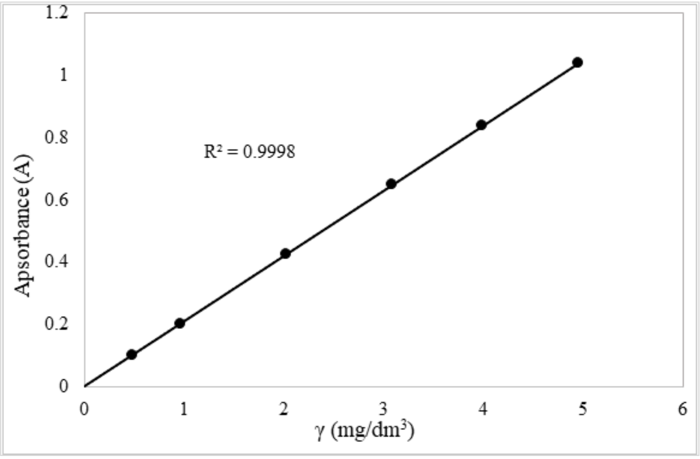

Figure S14. Calibration curve of MB dye

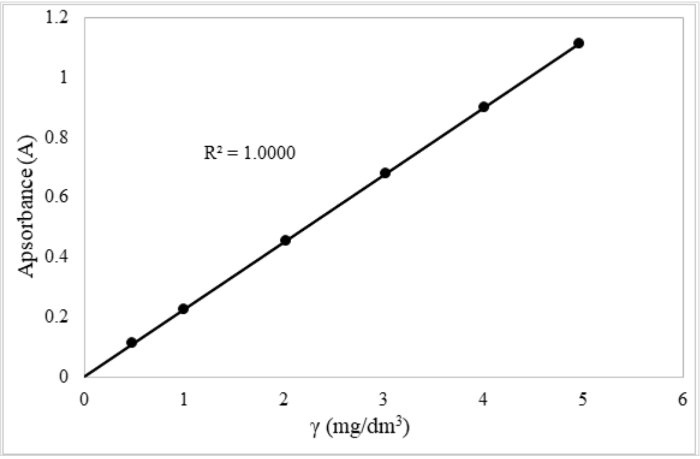

Figure S15. Calibration curve of RB dye

Structural analysis of zeolites

Table S3. Characterization of zeolites

Adsorbent	Chemical formula	Na_2O (%)	SiO_2/Al_2O_3	Sp (m^2/g)
NH_4BETA	$(NH_4,Na)_xO{\cdot}Al_2O_3{\cdot}25SiO_2{\cdot}4H_2O$	0.05	25.00	680
NH_4ZSM-5	$(NH_4, Na)_x O{\cdot}Al_2O_3{\cdot}30SiO_2$	0.05	30.00	400
NaY	$Na_{56}(Al_2O_3)_{28}(SiO_2)_{143}{\cdot}250H_2O$	13.00	5.10	900

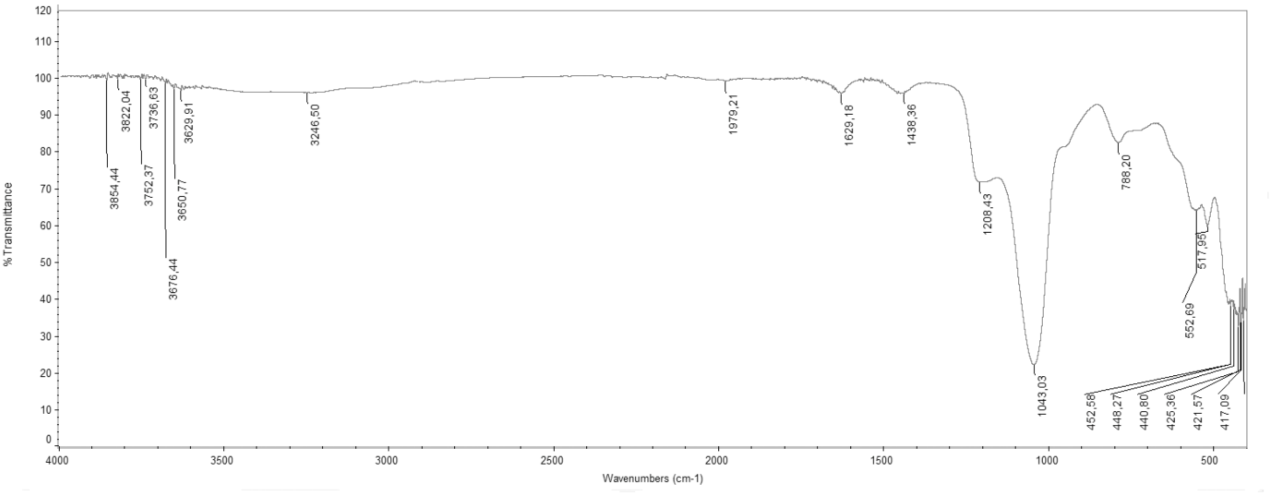

Figure S16. FT-IR spectra NH_4BETA zeolite

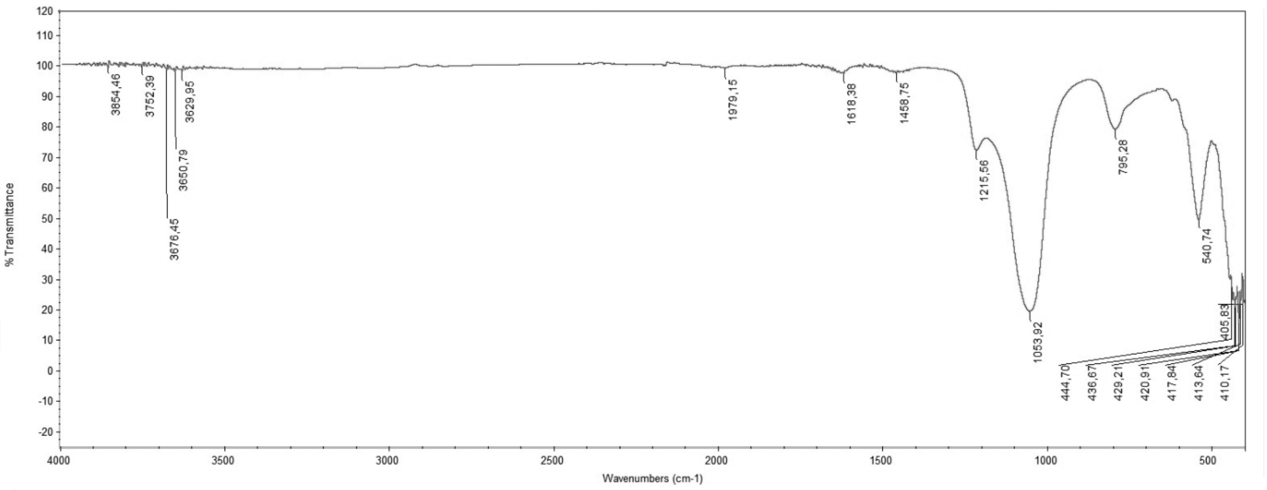

Figure S17. FT-IR spectra NH_4ZSM-5 zeolite

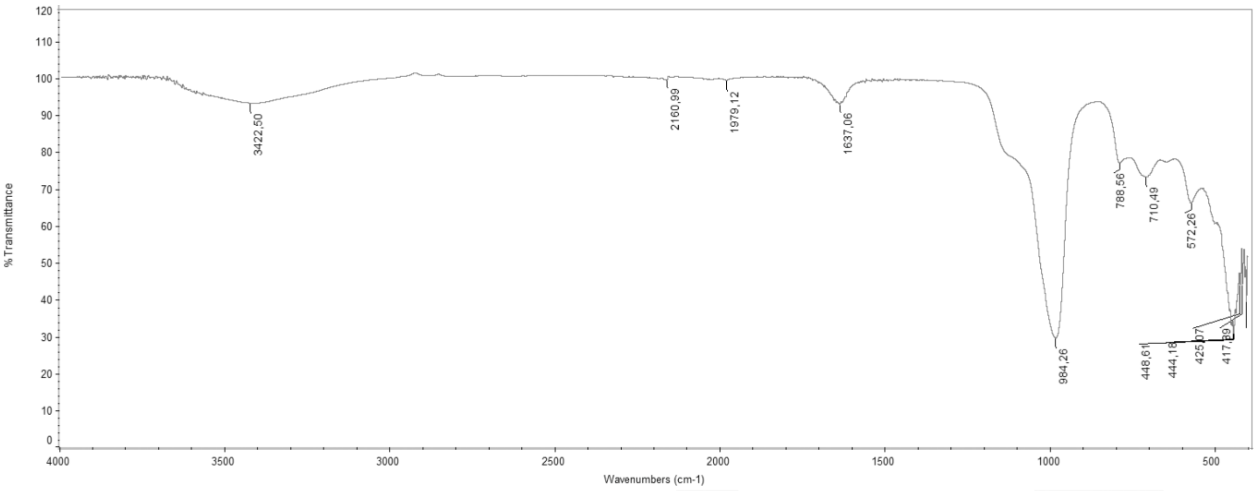

Figure S18. FT-IR spectra NaY zeolite

Analytical procedures

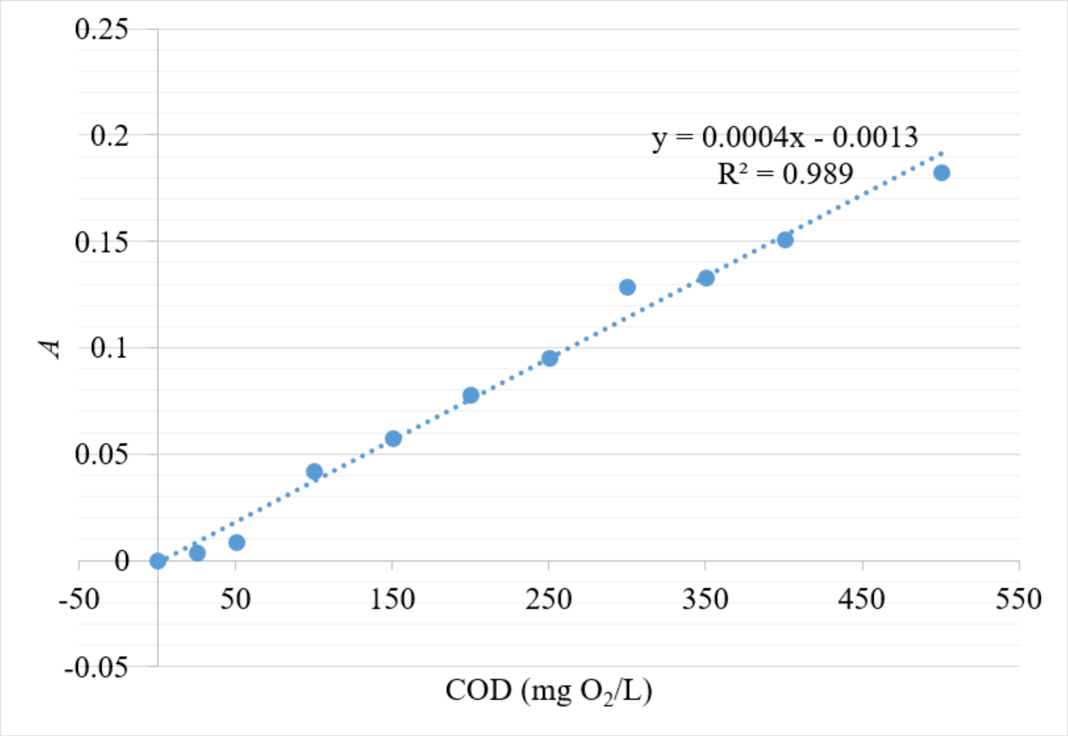

Figure S19. Calibration curve for determination of COD
